# Peripheral *SLC6A4* DNA Methylation Is Associated with *In Vivo* Measures of Human Brain Serotonin Synthesis and Childhood Physical Aggression

**DOI:** 10.1371/journal.pone.0039501

**Published:** 2012-06-20

**Authors:** Dongsha Wang, Moshe Szyf, Chawki Benkelfat, Nadine Provençal, Gustavo Turecki, Doretta Caramaschi, Sylvana M. Côté, Frank Vitaro, Richard E. Tremblay, Linda Booij

**Affiliations:** 1 Sainte-Justine Hospital Research Center, Montreal, Quebec, Canada; 2 Department of Pharmacology and Therapeutics, McGill University, Montreal, Quebec, Canada; 3 Sackler Program for Epigenetics and Psychobiology, McGill University, Montreal, Quebec, Canada; 4 Department of Psychiatry, McGill University, Montreal, Quebec, Canada; 5 Department of Psychiatry, University of Montreal, Montreal, Quebec, Canada; 6 Department of Psycho-Education, University of Montreal, Montreal, Quebec, Canada; 7 Department of Psychology and Pediatrics, University of Montreal, Montreal, Quebec, Canada; 8 School of Public Health, Psychotherapy and Population Sciences, University College Dublin, Dublin, Ireland; University of Texas, M.D. Anderson Cancer Center, United States of America

## Abstract

The main challenge in addressing the role of DNA methylation in human behaviour is the fact that the brain is inaccessible to epigenetic analysis in living humans. Using positron emission tomography (PET) measures of brain serotonin (5-HT) synthesis, we found in a longitudinal sample that adult males with high childhood-limited aggression (C-LHPA) had lower *in vivo* 5-HT synthesis in the orbitofrontal cortex (OBFC). Here we hypothesized that 5-HT alterations associated with childhood aggression were linked to differential DNA methylation of critical genes in the 5-HT pathway and these changes were also detectable in peripheral white blood cells. Using pyrosequencing, we determined the state of DNA methylation of *SLC6A4* promoter in T cells and monocytes isolated from blood of cohort members (N = 25) who underwent a PET scan, and we examined whether methylation status in the blood is associated with *in vivo* brain 5-HT synthesis. Higher levels of methylation were observed in both T cells and monocytes at specific CpG sites in the C-LHPA group. DNA methylation of *SLC6A4* in monocytes appears to be associated more reliably with group membership than T cells. In both cell types the methylation state of these CpGs was associated with lower *in vivo* measures of brain 5-HT synthesis in the left and right lateral OBFC (N = 20) where lower 5-HT synthesis in C-LHPA group was observed. Furthermore, *in vitro* methylation of the *SLC6A4* promoter in a luciferase reporter construct suppresses its transcriptional activity supporting a functional role of DNA methylation in *SLC6A4* promoter regulation. These findings indicate that state of *SLC6A4* promoter methylation is altered in peripheral white blood cells of individuals with physical aggression during childhood. This supports the relevance of peripheral DNA methylation for brain function and suggests that peripheral *SLC6A4* DNA methylation could be a marker of central 5-HT function.

## Introduction

It is important to study the interactions between epigenetic processes such as DNA methylation and human behaviours. This is a challenging task if we wish to establish a link between the behaviours in living human subjects and their epigenetic profile, as brain samples cannot be readily obtained for analysis. Therefore, alternative methods of epigenetic analysis must be identified. A logical approach will be to understand the relationship between expression of critical neurotransmitter systems in the brain and DNA methylation in cells of the relatively easily accessible peripheral system. Given the role of serotonin (5-HT) in a wide range of psychopathologies including impulsive aggression and recently reported associations between DNA methylation in 5-HT genes and depressive symptoms [1], [2], investigation of associations between DNA methylation in 5-HT genes and brain 5-HT function is of particular interest.

Observations in humans and animals have shown that aggressive behaviour is associated with low 5-HT neurotransmission [3], [4]. Using positron emission tomography (PET), we previously compared, in a longitudinal sample followed since childhood, *in vivo* brain 5-HT synthesis in healthy adult males with high and low levels of childhood physical aggression [5]. We found that adult males with high childhood-limited aggression (C-LHPA) had lower 5-HT synthesis in the left and right lateral orbitofrontal cortex (OBFC) relative to individuals with low aggression in childhood (LPA), even though there were no group differences in current behaviours or psychosocial functioning [5]. In the present study, we tested whether DNA methylation changes are involved in the long-term reduction of *in vivo* measures of 5-HT synthesis and associated childhood-limited aggressive behaviours. This was based on our previous DNA methylation studies showing that early life experiences result in stable changes in DNA methylation that affect long-term gene expression programming and the phenotype [6], [7], [8], and the hypothesis that the DNA methylation response to early life adversity is not necessarily limited to the brain [9].

Serotonin transporter (SLC6A4/5HTT) was selected as a candidate gene, given its crucial and widely demonstrated role in brain development [10], [11] and aggression [12], [13]. Also, preliminary data generated by genomic methylated DNA immunoprecipitation microarray (MeDIP-chip) using isolated T cells from the blood of the same cohort, indicated altered methylation at the *SLC6A4* gene promoter upstream of the transcription start site (TSS) in chronically aggressive individuals **(**Provençal, unpublished observations). Since DNA methylation patterns are cell-type selective [14], we isolated white blood cell types (T cells and monocytes) using immunomagnetic separation to reduce the complexity of the DNA methylation patterns. We quantified the state of DNA methylation of *SLC6A4* in T cells and monocytes isolated from blood of healthy males that were followed prospectively since childhood using pyrosequencing. The DNA methylation results were correlated with PET data from the same individuals to examine whether the state of DNA methylation in peripheral T cells and monocytes is associated with childhood-limited aggression and *in vivo* measures of brain 5-HT synthesis. We also explored the association between the widely investigated *SLC6A4* genotype (*ss*, *sl*, *ll*; [15]) and DNA methylation in the *SLC6A4* gene. Finally, to test whether DNA methylation plays a functional role in *SLC6A4* gene transcription, we subcloned a fragment of the *SLC6A4* promoter into a CpG-free pCpGL-basic luciferase reporter plasmid, *in vitro* methylated the promoter CpGs and measured luciferase activity in JAR cells that express *SLC6A4* endogenously.

## Results

### *SLC6A4* Methylation and Childhood-limited Aggression

**Figure 1:**
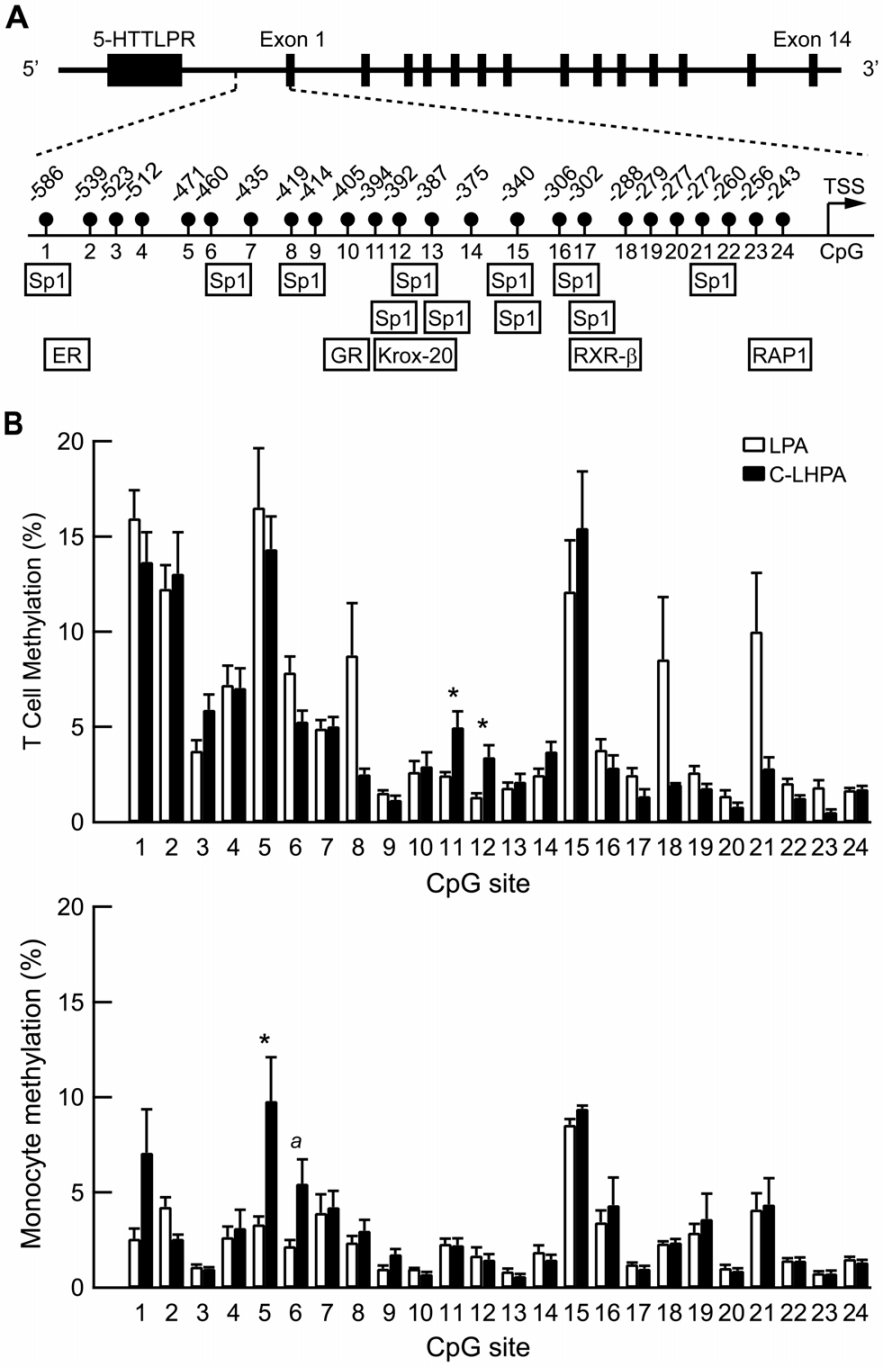
DNA Methylation of the *SLC6A4* promoter. (A) Schematic showing region of the *SLC6A4* promoter analyzed using sodium bisulfite pyrosequencing. The transcription start site (TSS) is indicated by the arrow. Positions of the 24 CpG dinucleotides are indicated as solid circles. Transcription factor binding sites predicted using Alibaba 2.1 are indicated as boxes below the CpG sites. (B) Upper panel represents mean±SEM percentage of DNA methylation of the C-LHPA (*n* = 8) vs. LPA (*n* = 17) in T cells. Lower panel represents DNA methylation percentage of the C-LHPA (*n* = 7) vs. LPA (*n* = 16) in monocytes. *p<0.05, ^a^ *p* = 0.06.

DNA methylation status of a 412 bp region upstream of the *SLC6A4* promoter containing 24 CpG sites was quantified using pyrosequencing in 25 individuals: 18 LPA and 7 C-LHPA (Figure 1a). This region is enriched with Sp1 binding sites, as well as other transcription factors such as ER, GR, Krox-20, RXR-beta and RAP1 (Figure 1a) as determined using TRANSFAC database (Alibaba 2.1).

#### T Cells

The mean percentage in DNA methylation across all the 24 CpG sites (±SE) for T cells was 5.31±0.5%. Differences in average methylation levels across all CpG sites between the C-LHPA and LPA group were not significant (4.31±0.5% and 5.77±0.7%, respectively, *p* = 0.25). A more detailed examination of DNA methylation levels at the level of the individual CpG sites showed high variability in percentage of methylation across the 24 sites, varying from 1.16±0.34% (CpG 20) to 15.88±3.91% (CpG 5). Group differences in percentage of methylation were observed at CpG sites 11 and 12 (Figure 1b). These CpG sites correlated highly with each other (*r* = 0.53, *p* = 0.008). Examining CpG site 11 and 12 conjointly in a multivariate general linear model (GLM), allowing to examine and correct for intercorrelations of CpG sites, showed a multivariate difference between the C-LHPA group and the LPA group (F(2,21) = 4.58, *p* = 0.02, partial η^2^ = 0.30)). The C-LHPA group had higher levels of *SLC6A4* methylation at CpG 11 (F(1,21) = 7.01, *p* = 0.01, partial η^2^ = 0.24) and at CpG12 (F(1,21) = 6.35, *p* = 0.02, partial η^2^ = 0.22) compared to the LPA group. When the average methylation levels in CpG sites 11 and 12 were combined, GLM showed that the group difference in average methylation levels across CpG site 11 and 12 was also significant (F(1,21) = 9.59, *p* = 0.005, partial η^2^ = 0.30).

#### Monocytes

The average percentage in DNA methylation across all the 24 CpG sites (±SE) for monocytes was 2.84±0.26%, with a higher percentage in overall methylation across the 24 CpG sites for the C-LHA group than for the LPA group (3.61±0.6% and 2.49±0.2%, F(1,21) = 4.47, *p* = 0.047, partial η^2^ = 0.18). As observed for T cells, there was considerable variability between CpG sites, varying from 0.77±0.08% (CpG 23) to 9.01±0.4% (CpG 15) (Figure 1b). Group differences in percentage of methylation were observed at CpG sites 5 and 6 (Figure 1b). These CpG sites correlated considerably with each other (*r* = 0.68, *p*<0.001). A multivariate analysis in which these CpG sites were examined conjointly showed a trend for the overall multivariate effect (F(2,20) = 2.61, *p* = 0.098, partial η^2^ = 0.21). Percentage of methylation was higher in the C-LHPA group than in the LPA group for CpG 5 (F(1,21) = 4.95, *p* = 0.04, partial η^2^ = 0.19) and tended to be higher for CpG 6 (F(1,21) = 3.79, *p* = 0.06, partial η^2^ = 0.15). When the average methylation levels in CpG sites 5 and 6 were combined, the group difference in average methylation levels across CpG site 5 and 6 was significant (F(1,21) = 5.48, *p* = 0.03, partial η^2^ = 0.21). There were no group differences in the other investigated CpG sites.

### Correlation of *SLC6A4* Methylation and *In Vivo* Brain 5-HT Synthesis

**Figure 2:**
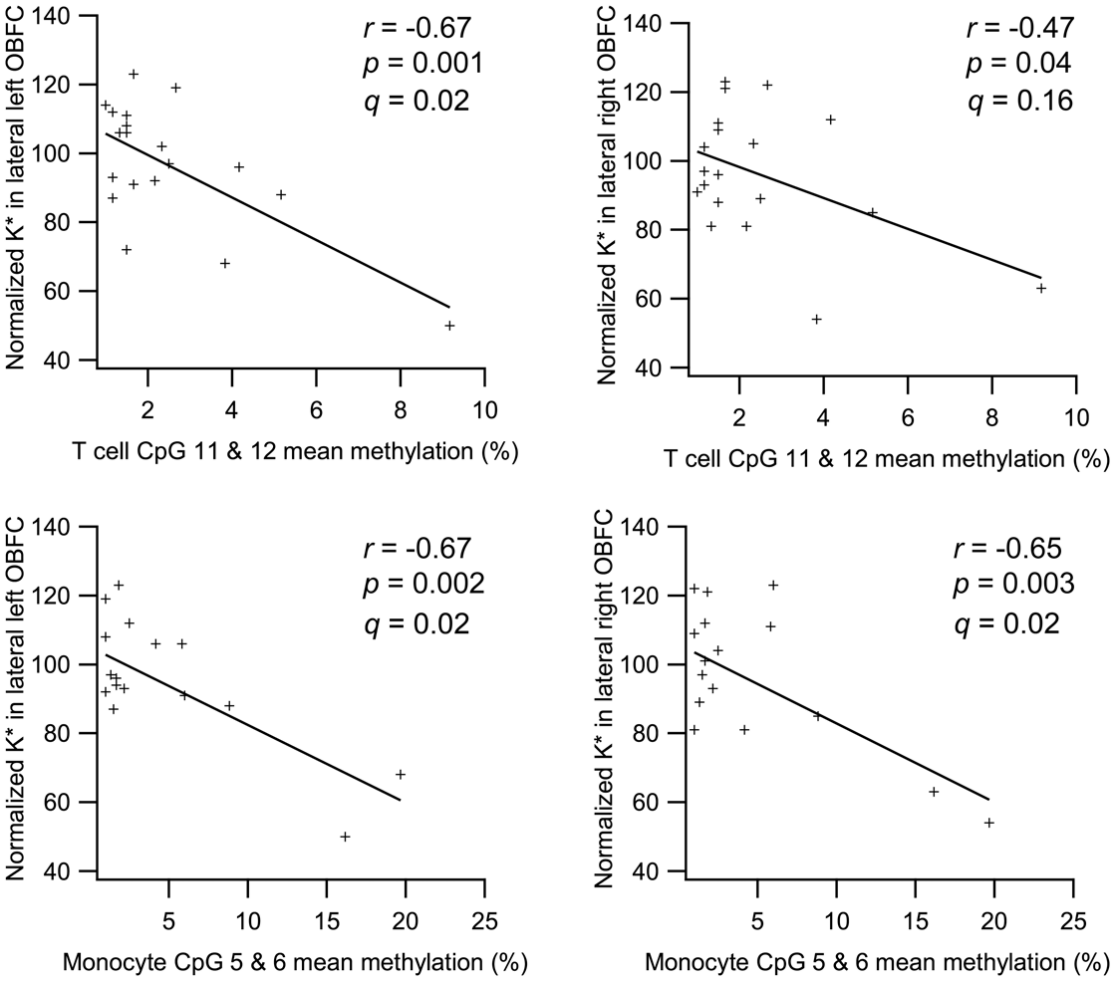
Correlations between peripheral methylation and brain 5-HT synthesis. In the upper panel, mean percentage of CpG site 11 and 12 methylation in T cells (*n* = 20) was negatively associated with the *in vivo* index of 5-HT synthesis (normalized K* value) in the lateral left (*r* = −0.67, *p* = 0.001) and right OBFC (*r* = −0.47, *p* = 0.04). FDA was used to adjust for multiple testing (*q* = 0.02 and *q* = 0.16, respectively). Bottom panel shows similar associations of percentage of methylation of CpG site 5 and 6 in monocytes (*n* = 19) vs. lateral left (*r* = −0.67, *p* = 0.002) and right OBFC (*r* = −0.65, *p* = 0.003). For both regions, *q* = 0.02.

We then examined whether the rate of 5-HT synthesis as determined by PET correlates with the state of *SLC6A4* methylation in T cells and monocytes. We focused on brain regions that have previously been associated with childhood-limited physical aggression [5], and on those CpG sites that were associated with childhood-limited aggression. Significant associations were found with the mean methylation of the CpG sites in *SLC6A4* promoter that were affected by aggression, CpG 11 and 12 for T cells (*n* = 20) and CpG 5 and 6 for monocytes (*n* = 19) respectively (Figure 2). Specifically, negative associations were observed between the mean methylation of CpG sites 11 and 12 in T cells and 5-HT synthesis in the lateral left (*r* = −0.67, *p* = 0.001) and right (*r* = −0.47, *p* = 0.04) OBFC of the brain. The first correlation remained significant after correction for multiple comparisons, while the second did not (*q* = 0.02 and *q* = 0.16, respectively). Significant correlations were found in CpG site 5 and 6 of monocytes as well, where higher mean methylation was associated with lower 5-HT synthesis in the lateral left (*r* = −0.67, *p* = 0.002) and right (*r* = −0.65, *p* = 0.003) OBFC regions. These correlations survived correction for multiple testing (*q* = 0.02 for both regions). No other voxels of interest (VOI) were associated with the *SLC6A4* methylation data, except that there was a trend of an association between increased DNA methylation in CpG sites 5 and 6 in monocytes and higher levels of brain 5-HT synthesis in the left hippocampus (*r* = 0.45, *p* = 0.056), although this association did not survive correction for multiple comparisons (*q* = 0.18). Nevertheless, the finding is consistent with a comparable trend observed between childhood-limited physical aggression and increased 5-HT synthesis in parts of the hippocampal areas observed in our previous study [5].

### *SLC6A4* Genotyping and *SLC6A4* DNA Methylation

A polymorphic region located in the promoter of *SLC6A4* (5-HTTLPR) is known to affect gene expression, where the long *l* allele results in higher *SLC6A4* transcription than the shorter *s* variant [15], [16]. We had genetic information for several subjects (*ss*, *n* = 9; *sl*, *n* = 7; *ll*, *n* = 4), and found no relationships between mean methylation levels and 5-HTTLPR genotype, neither in T cells nor monocytes (*p*>0.10).

### Promoter *SLC6A4* Methylation Alters Gene Expression

**Figure 3:**
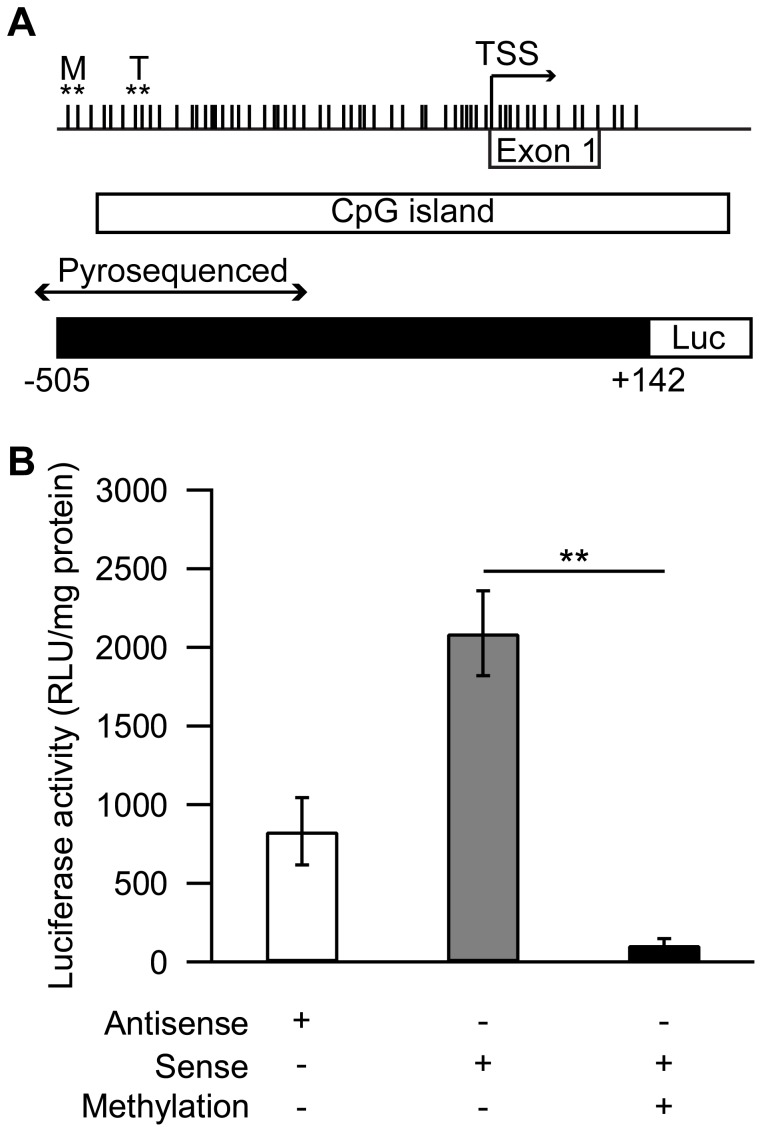
Methylation regulates gene expression at *SLC6A4* promoter. (A) Schematic representation of the 648 bp *SLC6A4* promoter construct subcloned into the pCpGL-basic plasmid. CpG sites are indicated as vertical lines on the gene promoter, the significantly altered CpG sites in both T cells and monocytes from pyrosequencing were included in the construct. The TSS, exon 1 and the CpG island locations within the *SLC6A4* promoter are also shown in the figure. (B) Normalized luciferase activity (RLU/mg protein) in the JAR cell line for the antisense, sense, and methylated sense promoter constructs are shown. Values are expressed as mean±SEM. **p<0.01.

Lastly, we generated a 648 bp CpG-free luciferase reporter plasmid and performed *in vitro* methylation with SssI DNA methyltransferase to determine whether *SLC6A4* promoter methylation can affect its transcriptional activity (Figure 3a). DNA methylation significantly decreased the normalized luciferase activity (*p* = 0.008), suggesting that methylation of the CpG sites could potentially play a role in silencing the transcriptional activity of this promoter region (Figure 3b). We also examined the mRNA expression of *SLC6A4* in T cells using reverse transcription polymerase chain reaction (RT-PCR). However, levels of mRNA were very variable between individuals and therefore no significant differences of *SLC6A4* expressions were observed between the high and low aggression trajectories (*n* = 13).

## Discussion

The main challenge in addressing the role of DNA methylation in human behaviour is the fact that the brain is inaccessible to epigenetic analysis in living humans. DNA methylation patterns are, to a large extent, tissue specific [14]. Therefore, it is anticipated that only a fraction of DNA methylation events will show correspondence between the periphery and the brain. The present study has tested, for the first time, correlation of peripheral white blood cell DNA methylation states and brain imaging of 5-HT synthesis in healthy adult males with different levels of childhood physical aggression. These individuals were previously shown by us to express lower levels of 5-HT in the brain. This suggests a long-term effect on gene expression in the brain of the events that were associated with aggression during childhood. We therefore took advantage of this study to determine whether changes in brain 5-HT synthesis were associated with changes in DNA methylation in T cells and monocytes. We found significantly higher *SLC6A4* promoter DNA methylation in specific CpG sites in T cells and monocytes isolated from the C-LHPA group compared with the LPA group. In monocytes, in addition to increased methylation at specific CpG sites, we observed an increase in the average DNA methylation across the CpG sites in the pyrosequenced region. Notably, the T cell DNA methylation state is less reliably associated with group membership (aggression) than the monocyte methylation patterns that show overall multivariate associations with group. There is an ongoing debate on what is the best source for peripheral information on DNA methylation for behaviour and brain studies [17]. Our study provides a preliminary indication for using monocytes as a more reliable source. However further studies comparing different peripheral cell types are required before we could conclusively address this issue.

Higher mean methylation of particular CpG sites was associated with lower *in vivo* measures of 5-HT synthesis in the left and right lateral OBFC, demonstrating an association between peripheral white blood cell DNA methylation and brain 5-HT synthesis. The association between *SLC6A4* and 5-HT synthesis is supported by other studies. Previous studies have shown that 5-HT depletion results in reduced *SLC6A4* expression [18]. *SLC6A4* knockout mice have altered brain 5-HT synthesis and concentration, suggesting that *SLC6A4* plays a role in brain 5-HT function [19]. Interestingly, the differentially methylated CpG 6, 11, and 12 were also found to be differentially methylated in another study that focused on maternal depression [1]. Although we were able to correlate *SLC6A4* methylation with 5-HT synthesis in the brain, we did not observe differences in *SLC6A4* expression in blood cells. We did not find *SLC6A4* genotype and mRNA to be associated with aggression and mean methylation. Due to small sample size, it was not possible to test for interaction between genotype and methylation status on mRNA levels. Therefore, it is uncertain whether the differential DNA methylation that we observed is independent of genetic variation. The variability in the mRNA levels may be caused by low extraction yield of intact RNA due to sensitivity to processing time [20]. In contrast, DNA is more robust and methylation remains more constant over time, thus supporting the conclusion that DNA methylation in peripheral cells is a more reliable measure of central 5-HT synthesis [21].

Higher levels of *SLC6A4* methylation at specific sites in T cells and monocytes were specifically associated with lower *in vivo* brain 5-HT synthesis in the lateral left and right OBFC. This is of particular interest because these specific brain regions also demonstrated lower 5-HT synthesis in the C-LHPA trajectory [5]. DNA methylation differences in *SLC6A4* gene cannot directly explain 5-HT synthesis in the brain and other mechanism must be mediating between changes in *SLC6A4* programming and 5-HT synthesis. Nevertheless, *SLC6A4* methylation from blood derived cells was previously associated with psychiatric disorders such as depression and alcoholism in humans [1], [2]. Furthermore, *SLC6A4* has been used as a peripheral marker for various neuropsychiatric disorders that involve 5-HT alterations [22], [23], [24].

The luciferase reporter construct has demonstrated a functional role of DNA methylation on *SLC6A4* gene expression. Variable levels of methylation across the *SLC6A4* promoter are observed *in vivo*. *In vitro* methylation of the *SLC6A4* promoter including the CpG sites associated with aggressive behaviour was able to reduce transcriptional activity, suggesting that DNA methylation of CpG sites within this region silences *SLC6A4* promoter activity. Using Alibaba 2.1 program, a list of potential transcription factors that may interact with the CpG sites was generated. Unquestionably, future studies are needed to examine the promoter more in depth to determine whether *SLC6A4* expression is regulated by specific CpG sites and whether there are transcription factor interactions at specific regions of the promoter. Nevertheless, these findings, together with the present observed associations between *SLC6A4* methylation and 5-HT synthesis and childhood-aggression, may suggest that methylation is silencing *SLC6A4* expression in certain cells in the periphery and perhaps also the CNS sometimes during development, as a result of early stress, thereby disrupting the developmental regulation/ontogeny of the 5-HT system.

This pilot study has some limitations. First, the participants were limited to healthy males. It would be of interest to see to what extent the results can be generalized to females. Secondly, although the sample is comparable with other PET studies, the sample was too small to reliably investigate interactions between aggression, 5-HT synthesis and DNA methylation. Third, the methylation analysis was only done on peripheral tissue and the state of DNA methylation in the brain was not available. Although it is not possible to obtain human brain tissue from living individuals, this type of analysis can be replicated in nonhuman primates. Fourth, the blood and PET data from the participants were collected during adulthood and the data obtained was then associated with the assessments of their childhood aggressive behaviour. It will be important to obtain brain functioning and DNA methylation states during childhood, adolescence and adulthood to fully monitor the development and progression of DNA methylation of *SLC6A4* and brain 5-HT synthesis with the development of physical aggression. The peripheral DNA methylation biomarker discovered in our study makes this repeated measure feasible. Lastly, we are aware that changes in *SLC6A4* promoter methylation and brain 5-HT function may be due to factors other than aggression. However, in order to minimize confounders the participants in this study were carefully selected based on their childhood phenotype and were carefully screened for presence of any detectable current mental or physical health problems.

Taken together, this study is among the first to associate peripheral methylation of the *SLC6A4* gene in the blood and *in vivo* measures of brain 5-HT function. This observation strongly supports the relevance of DNA methylation states in peripheral cells for brain function. The combination of higher *SLC6A4* methylation and lower 5-HT synthesis in the brain suggests vulnerability for impulsive behaviour in subjects with high childhood-limited aggression. More importantly, the observed differential *SLC6A4* methylation detected in peripheral blood cells suggests the feasibility of using methylation at specific CpG sites of *SLC6A4* as non-invasive biomarkers of 5-HT synthesis and behaviours associated with altered 5-HT function such as aggression. If these results can be confirmed in a larger sample, methylation of *SLC6A4* CpG sites could be used to screen for 5-HT related psychiatric disorders as well as for the assessment of preventive and corrective interventions.

## Materials and Methods

### Participants

Ethical approval was granted by the research committee at Sainte-Justine Hospital Research Center and written informed consents were obtained from all participants. Healthy adult males (mean age±SD: 27.1±0.7) were selected from a 21 year longitudinal study with low socioeconomic status French-speaking families in a disadvantaged area of Montreal (N = 1,037) [25]. The participants were followed since kindergarten with evaluations of physical aggression made by teachers at age 6 and annually between ages 10–15 with the Teacher Form of a French-Canadian version of the Social Behaviour Questionnaire [25]. Based on the measurements collected from childhood until age 15, four different trajectories for physical aggression were previously identified for the entire sample of the longitudinal study (N = 1,037 [25]). One trajectory showed no physical aggression at any time points (never); a second trajectory demonstrated low to moderate aggression and declined between age 10–15 (low level desister); a third trajectory demonstrated high rates of aggression, which subsequently declined over time (childhood-limited high physical aggression (C-LHPA)); a final trajectory consisted of individuals with consistently high physical aggression until age 15 (chronic). Chronic aggressive subjects were not included in our study for the following reasons. First, our current study compared DNA methylation with imaging data that was derived from our previous brain 5-HT synthesis imaging study. That specific study compared healthy individuals who were aggressive in childhood but desisted in adolescence to the low level aggression group (See Booij et al. for more details [5]). Second, the chronic aggressive group is a small minority in the population that only represents approximately 3% of the entire cohort [25]. In this particular study, we wanted to focus on the majority of the population, which belongs to the other three trajectories. Finally, in the sample we tested, we aimed to limit the number of confounding factors that could have influenced the outcome measures, such as drug use, current psychopathology etc. Including chronic individuals in the PET study sample would have been confounded by the heterogeneous nature of the chronic aggressive group as they are very likely to differ also on several critical confounders. In this study the participants from C-LHPA (*n* = 8) were compared with the low physical aggression (LPA) trajectories consisted of never and low level desister groups (*n* = 17) [5]. Twenty of the subjects were previously involved in a study investigating childhood physical aggression and brain 5-HT synthesis where their [^11^C]AMT PET data have been collected [5]. Briefly, the participants were each given an intravenous injection of 10 mCi (370 MBq) of α-[^11^C]AMT after psychiatric interview [26] and medical evaluation, then underwent a 60 min dynamic PET scan using an ECAT HR+ (CTI Molecular Imaging Inc/Siemens). Furthermore, genotyping data of the *SLC6A4* promoter polymorphism (5-HTTLPR) for these participants were obtained by polymerase chain reaction (PCR) method as previously described [27]. Owing to technical problems, two monocyte samples, one from each group were dropped for the pyrosequencing analysis. Moreover, for the C-LHPA group, one monocyte sample was missing data on CpG 5 and one T cell sample was missing data on CpG 1–14.

### Lymphocyte Extraction and Sample Preparation

Peripheral blood samples were collected from participants and stored in EDTA coated tubes at 4°C before extraction. Peripheral blood mononuclear cells (PBMC) were isolated by density gradient centrifugation with Ficoll-Paque (GE Healthcare). Briefly, blood was mixed with PBS (GIBCO, Invitrogen) and transferred into a tube with Ficoll-Paque. After 30 min of centrifugation at 2000 rpm at 18°C, the PBMC layer was extracted, washed twice with HBSS (GIBCO, Invitrogen) and resuspended in PBS/FBS (PBS with 4% FBS). T cell extraction was done by immunomagnetic separation with Dynabeads CD3 (Dynal, Invitrogen) and EasySep Magnet (Stemcell Technologies). Dynalbeads were agitated and washed twice with PBS/FBS with the magnet, and incubated with the PBMCs on a nutator at 4°C for 45 min. CD3+ T cells were separated from the PBMCs with the magnet and washed 3 times with PBS/FBS. CD19+ B cells were also isolated with Dynabeads CD19 Pan B (Dynal, Invitrogen) and the left over cells consisted of monocytes. For the purpose of this study, T cells and monocytes were used for the methylation analysis. After isolation, the cell pellets were stored at −80°C until further processing. DNA and RNA extraction were performed using Trizol (Invitrogen) according to the manufacturer’s instructions and stored at −20°C and −80°C respectively. RNA qualities were analyzed using Agilent 2100 Bioanalyzer done by Genome Quebec.

### Pyrosequencing

To investigate the DNA methylation pattern in the target region 214–625 bp upstream of the *SLC6A4* gene promoter, three sets of outside primers and four sets of nested primers were used as follows: Out F1&2 5′-TGTAGTTGGTTAATAAAATGAGAATTAGTT-3′, Out R1&2 5′-AAATCCTAACTTTCCTACTCTTTAACTTTA-3′, Out F3 5′-TTTTAGGAAGAAAGAGAG-AGTAGTTTT-3′, Out R3 5′-CCAAAAAACTCTTAAAAAATTTTTAC-3′, Out F4 5′-TTTGT-TTTTTTGTGTAGTTTTTTTT-3′, Out R4 5′-CTCACATAATCTAATCTCTAAATAACC-3′, Nest F1 5′-TTTTTTATTGTGGAAGTTTTTATTGTG-3′, Nest R1 5′-CTCTCTCTTTCTTCCT-AAAACCTAACA-3′, Nest F2 5′-TTGTTAGGTTTTAGGAAGAAAGAGAGA-3′, Nest R2 5′-AAAAAAAACTACACAAAAAAACAAATATAC-3′, Nest F3 5′-TTTTAGGAAGAAAGAG-AGAGTAGTTTT-3′, Nest R3 5′-AAATCCTAACTTTCCTACTCTTTAACTTTA-3′, Nest F4 5′-TAAAGTTAAAGAGTAGGAAAGTTAGGATTT-3′, and Nest R4 5′-ACCCCAAAACCA-AAAAAAAA-3′. The nested reverse primers were biotinylated for pyrosequencing. DNA was treated with sodium bisulfite and two rounds of PCR amplification was performed as previously described [8]. 15 µl of the PCR products were used to perform pyrosequencing using PyroMarkQ24 (Qiagen) according to the manufacturer’s protocol. The methylation percentage at each CpG site was analyzed using the PyroMark Q24 software (Qiagen).

### Luciferase Reporter Construct

To generate a 648 bp construct from the *SLC6A4* promoter, outside primers were adapt from a previous study [28], and nested primers were designed as follows: Sense Fow 5′-TTGGATCCT-GCTAGGTCCCAGGAAGAAA-3′, Sense Rev 5′-TTAAGCTTGGGGAAGAAGGTCTGGAA-AG-3′, Antisense Fow 5′-TTAAGCTTTGCTAGGTCCCAGGAAGAAA-3′, and Antisense Rev 5′-TTGGATCCGGGGAAGAAGGTCTGGAAAG-3′. Human genomic DNA was subjected to two rounds of amplification with Failsafe PCR Enzyme Mix (Epicentre) with the outside and nested sense and antisense primers, digested with HindIII and BamHI and subcloned into the CpG-free pCpGL**-**basic luciferase reporter plasmid. To create methylated constructs, SssI DNA methyltransferase (New England Biolabs) was used for *in vitro* methylation [29]. Using this strategy, CpGs in the promoter are exclusively methylated with no confounding effect of methylation of the surrounding plasmid sequence. Both the methylated and non-methylated plasmids were then transfected into JAR human placental choriocarcinoma cells (ATCC HTB-144) that constitutively expresses *SLC6A4* [30], [31] using standard calcium phosphate transfection method as previously described [32]. JAR cells were cultured in RPMI 1640 (GIBCO, Invitrogen) containing 10% fetal bovine serum (GIBCO, Invitrogen), plated at a density of 7 × 10^5^ cells/well in six-well plates and co-transfected with 200 ng of plasmid DNA. Cells were harvested and lysed 48 h after transfection and luciferase activity was measured using the Luciferase Assay System (Promega).

### VOI Analyses

The imaging data were pre-processed, generated and normalized as described previously [5]. Next, VOI were identified as follows: hippocampus, lateral and medial OBFC, and caudate. These VOIs were selected based on their prominent role in emotion regulation and involvement with aggression phenotypes. Primary VOI for the purpose of this study was the lateral OBFC, based on our results of our previous study [5]. Associations between DNA methylation and the other regions were analyzed for exploratory purposes.

### *In silico* Transcription Factor Binding Analysis

Prediction of potential transcription factor binding sites from −240 to −590 of the *SLC6A4* promoter was performed using Alibaba 2.1 program (http://www.gene-regulation.com/pub/programs/alibaba2/index.html) with the following parameters: pairism to known sites 50, matrix width 10 bp, minimum number of sites 4, minimum matrix conservation 80%, similarity of sequence to matrix 1%, and factor class level 4.

### Statistical Analysis

Univariate and multivariate GLM were used to explore associations between trajectories of aggression and methylation. For all of these GLM, participants were statistically weighted according to the posterior probability of belonging to the assigned trajectory [5], [25]. These analyses were not corrected for multiple comparisons since these analyses were exploratory and primarily aimed to select specific CpG sites for the more detailed investigation of our primary research question about the association between peripheral DNA methylation and *in vivo* measures of brain 5-HT synthesis. Pearson’s correlations were used to assess associations between DNA methylation and brain measures. The false discovery rate (FDR) was used to adjust for multiple testing in these latter correlational analyses, using an adjusted q value of 0.05 [33]. Data were log10 transformed when required in order to meet the normality assumption for GLM, however original values are presented throughout the paper to facilitate interpretation of the data. For some of the CpGs there was some heterogeneity of variances but given the sample size we continued using parametric tests. Outliers and influential data points were investigated by examination of the absolute values of standardized residuals (>3) and Cooks distance (cutoff >1). No outliers or influential data points were detected in average levels in methylation across the 24 CpG sites in T cells or monocytes, neither in methylation levels in the specifically investigated CpG sites that were of further investigation (T cells: CpG 11 and 12, monocytes: CpG 5 and 6). Student’s t-test was used to compare gene transcriptional activation of the luciferase reporter construct. The significant threshold is set at *p*<0.05 and is indicated by asterisks.
